# The properties of fibreboard based on nanolignocelluloses/CaCO_3_/PMMA composite synthesized through mechano-chemical method

**DOI:** 10.1038/s41598-018-23497-x

**Published:** 2018-03-23

**Authors:** Yipeng Chen, Tailong Cai, Baokang Dang, Hanwei Wang, Ye Xiong, Qiufang Yao, Chao Wang, Qingfeng Sun, Chunde Jin

**Affiliations:** 1 School of Engineering, Zhejiang A & F University, Hangzhou, Zhejiang Province 311300 PR China; 2 Key Laboratory of Wood Science and Technology, Hangzhou, Zhejiang Province 311300 PR China

## Abstract

The purpose of this study was to develop a rapid and green method for the synthesis of lignocelluloses-based materials with superior mechanical properties. Samples were produced by hot-pressed method using different concentrations of CaCO_3_ and poly (methyl methacrylate) particles-filled nanolignocelluloses composites which was synthesized through mechano-chemical method. Poly (methyl methacrylate) and CaCO_3_ nanoparticles have been used as nanofillers. Bending strength, elasticity modulus, and dimensional stability, thermal properties of the developed lignocelluloses-based composites were determined. In view of the experimental results, it is found that the composites materials have good mechanical, dimensional stability, and thermal properties which enhanced as the filler loading increased. Thus, herein described lignocelluloses-based materials showed important characteristics to be concluded that these composites are suitable to be used for the design of flooring and construction systems.

## Introduction

It is widely known that the main components of lignocelluloses are hemicelluloses, lignin, and cellulose, which has been gaining acceptance in commodity applications, particularly in the construction sector, due to their easy processability and low maintenance costs^1–5^. More attention has been paid to the research of lignocellulose-based composites such as the lignocellulose-plastic composites^6–10^, lignocellulose/inorganic nanoparticles composites^11–13^, fiber/organic phase composites^14–18^. However, there have been only few literature reporting on the synthesis of high mechanical properties slab-like nanolignocellulose/inorganic composites. It is thought that nanoparticles could dramatically induce an improvement in mechanical and electrical properties, heat resistance, radiation resistance^19^, and other properties as a result of the nanometric scale dispersion of the filler in the lignocellulose matrix^11^. These composites generally exhibit superior stiffness, strength, and heat distortion temperatures with respect to unreinforced polymers^20^. Nanoparticles such as nanometric CaCO_3_^21^, SiO_2_^22^, Fe_3_O_4_^23^, TiO_2_^24^, and ZnO^25^ particles have been used to prepare nanocomposites. Among the various reinforcing materials, CaCO_3_ is attractive because of its low cost. Many efforts have been devoted to surface-treated CaCO_3_ filler to increase the interaction between the lignocellulose and filler. The effects of surface modification on mechanical properties have been positive^26^. The use of nano-CaCO_3_ particles may bring new insights in the study of lignocellulose–inorganic nanocomposites.

Through mechano-chemical synthesis, lignocellulose and nano-CaCO_3_ are mixed in certain proportion, running in the colloid grinder for a long time, the mechanical energy transfers to the lignocellulose. Under the repeated collision of grinding medium, lignocellulose effect by the action of impact, shear, compression and friction force, through repeated extrusion, grinding and cold welding process, forming high density dislocations calcium carbonate/lignocelluloses composites in this process. At the same time, the lignocelluloses are gradually refined to nanometer scale, and become ultrafine particles with dispersed distribution, which provides a fast channel for the mutual diffusion of atoms, thus obtaining the synthetic products^27^.

At present, the adhesives used in wood-based panel industry are widely used, such as urea formaldehyde resin, phenolic resin and melamine formaldehyde resin. The three kinds of adhesives are made of formaldehyde as raw material. Formaldehyde is regarded as one of the main sources of indoor air pollution and the pollution cycle is long and difficult to remove^28^. Here we mixed organic phase (poly (methyl methacrylate), PMMA) in our composites, which instead of adhesives as the reinforcement elements. This can be considered to have important potential as an alternative material for green composite.

Herein, we reported mechano-chemical and hot-pressed method for the synthesis of nanolignocellulose (NLC)/CaCO_3_/PMMA composites. The objective of the research was to characterize the mechanical properties and water resistance of lignocelluloses-based materials made from NLC/CaCO_3_/PMMA composites. The influence of different concentrations of CaCO_3_ and PMMA on the dimensional stability, thermal properties, and mechanical properties of the composites has been investigated.

## Results

**Figure 1:**
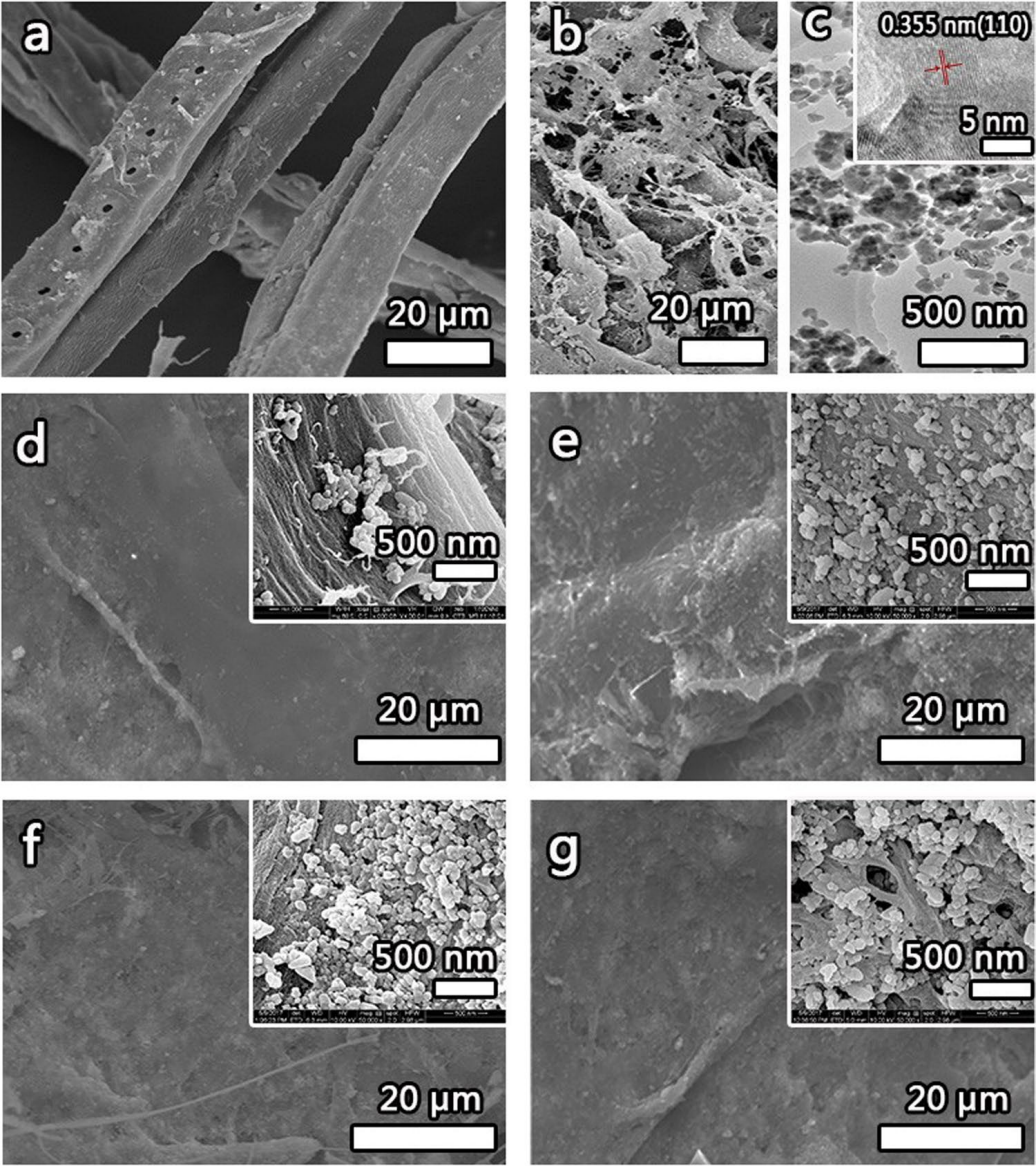
SEM image of untreated lignocellulose (**a**), the NLC/CaCO_3_/PMMA composites with 8 wt.% CaCO_3_ and 6 wt.% PMMA (**b**), the TEM image of the NLC/CaCO_3_/PMMA composites with 8 wt.% CaCO_3_ and 6 wt.% PMMA (**c**), and hot-pressed NLC/CaCO_3_/PMMA composites with different content of CaCO_3_ from 2–11% (**d**–**g**).

The morphology of the samples were investigated by SEM and TEM images, as showed in Fig. 1. Figure 1a showed the structure of the pit on the pine tube wall, and the average diameter of the lignocellulose was about 20 μm, and the length was hundreds of microns to millimeters. The smooth surface made it difficult to bond between the lignocellulose. As showed in Fig. 1b, the surface of the untreated lignocellulose after mechano-chemical processing was very rough, and the length and diameter of the lignocellulose were significantly reduced compared with that before the mechano-chemical processing. At the same time, the specific surface area of the lignocellulose and the probability of hydrogen bonding were significantly increased. As shown in the Fig. 1c, the NLC/CaCO_3_/PMMA composite with 8 wt.% CaCO_3_ and 6 wt.% PMMA typically had diameters of 100–500 nm. The HRTEM (inset of Fig. 1c) images showed a [001] zone-axis HRTEM image of this plate with a clearly resolved lattice fringe of the (110) planes (d = 0.355 nm)^29^, further confirming the single-crystalline nature of each vaterite plate and their size distributions showed that the mean diameters and standard deviation of CaCO_3_ were about 5.52 ± 1.72 nm. The SEM images of the samples synthesized with different content of CaCO_3_ were shown in Fig. 1d–g. One can see that the CaCO_3_ grow on the surface of NLC. Magnified micrographs of the composites were shown in the illustration. When the content of CaCO_3_ was 2% the spheral CaCO_3_ nanoparticles were obtained. When the content of CaCO_3_ increased to 11% (Fig. 1g), the amount of CaCO_3_ nanoparticles precipitated on the NLC surface was obviously increased. The inset of Fig. 1g noted that the NLC/CaCO_3_/PMMA composite was composed of randomly entangled nanowires and one can see that the CaCO_3_ nanoparticles grew on the surface of the NLC nanowires and CaCO_3_ nanoparticles attached to or partly embedded in the NLC nanowires.

**Figure 2:**
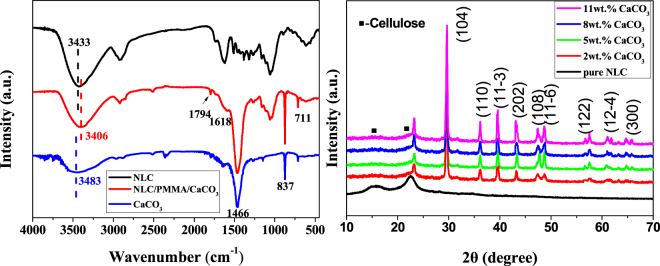
FTIR (**a**) spectra of the NLC and the NLC/CaCO_3_/PMMA composites and XRD (**b**) spectra of the NLC and the NLC/CaCO_3_/PMMA composites with different content of CaCO_3_ from 2–11%.

Figure 2a exhibited the FTIR spectra of the NLC, pure CaCO_3_ and the NLC/CaCO_3_/PMMA composites. The absorption bands at 3406, and 1618 cm^−1^ corresponded to the bands of the O–H bond, and the carbonyl group bending vibration, respectively. Most of the peaks represent major cell wall components in the NLC such as cellulose (1166, 891 cm^−1^), hemicelluloses (1738, 1116, 1051 cm^−1^) and lignin (1597, 1501, 1238 cm^−1^)^30,31^. The absorption peak of the NLC/CaCO_3_/PMMA composites at 1466 cm^−1^ was due to the ν_3–3_ CO_3_^2−^ and ν_3–4_ CO_3_^2−^ ^32^. Furthermore, one can clearly see the characteristics of calcite at 711 cm^−1^ and 873 cm^−1^, further indicated the existence of CaCO_3_ the NLC/CaCO_3_/PMMA composites^32^. The peak at 1794 cm^−1^ represented the C=O stretching mode of the ester group in the PMMA^33^. In addition, the absorption bands of the O–H bond of the NLC/CaCO_3_/PMMA composites shift to 3406 cm^−1^ compared with that of NLC (in 3433 cm^−1^) was attributed to a strong interaction between the hydroxyl groups of NLC and PMMA through hydrogen bonds. In Fig. 2b, two XRD diffraction peaks located at around 15° and 22° were assigned to the crystalline region of cellulose^34,35^. As for the NLC/CaCO_3_/PMMA composites, all the other diffraction peaks were indexed to well crystallized calcite with a hexagonal structure (JCPDS 47–1743)^36^. When the CaCO_3_ concentrations were to 2 wt.%, 5 wt.%, 8 wt.% and 11%, only a broaden peak at 2θ = 22.18° was observed, which may have resulted from the interaction between cellulose and inorganic materials or the overlapping resulted in the broaden peak at around 22.18°. The NLC/CaCO_3_/PMMA composites displayed more strong peak intensities with the increasing of the content of CaCO_3_.

**Figure 3:**
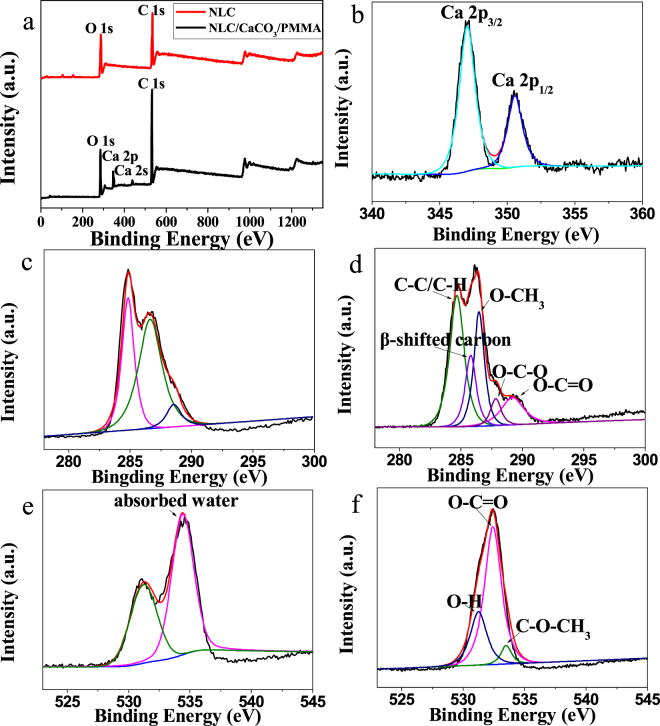
XPS spectra of (**a**) survey spectrum, (**b**) Ca 2p in NLC/CaCO_3_/PMMA composite and (**c**,**d**) C1s, (**e**,**f**) O1s in pure NLC and NLC/CaCO_3_/PMMA composite, respectively.

A typical XPS wide-scan spectrum from calcite was shown in Fig. 3a. NLC and the NLC/CaCO_3_/PMMA composites exhibited two major peaks with binding energy 285.0 and 531.3 eV corresponded to the C 1 s and O 1 s of cellulose, respectively. However, in the NLC/CaCO_3_/PMMA composites, an additional peak was observed at a binding energy of 346.9 and 437.6 eV corresponded to the Ca 2p and Ca 2 s of CaCO_3_. Figure 3b showed the high-resolution XPS spectra of the Ca (2p) core levels from the NLC/CaCO_3_/PMMA composites. Two peaks at 347.07 eV were due to Ca 2p_3/2_, and 350.6 eV was due to Ca 2p_1/2_^37^. This further confirmed the presence of CaCO_3_ in the NLC/CaCO_3_/PMMA composites. In order to reveal the chemical structure one should acquire a high-resolution spectrum of C1s (Fig. 3c and d). The C 1s envelope of the NLC/CaCO_3_/PMMA composites can be resolved into four components: hydrocarbon (C–C/C–H) at a binding energy of 284.7 eV, β-shifted carbon (due to their juxtaposition to O–C=O groups) at 285.7 eV^38^, methoxy groupcarbon at 286.4 eV and carbon in the ester group and carbonate:CO_3_ at 289.3 eV^37^, while the third one which appears as a shoulder at about 287.9 eV corresponded to the O–C–O bond. Figure 3f shows the fitted high resolution O1s spectra of NLC/CaCO_3_/PMMA composites. The O1s peak at a binding energy of 532.4 eV was assigned to carbonyl oxygen of the methyl esters groups of PMMA and of the –COOH. The O1s at a binding energy of 533.5 eV corresponded to bridge oxygen atoms C–O–CH_3_ of the methyl ester group of PMMA^39^. The fitting peak at around 531.2 eV was attributed to oxygen in OH groups which suggested that PMMA obtained on NLC surface via the OH–bond. However, the presence of the peak of NLC at 533.5 eV was attributed to the multiplicity of the adsorbed water.

**Figure 4:**
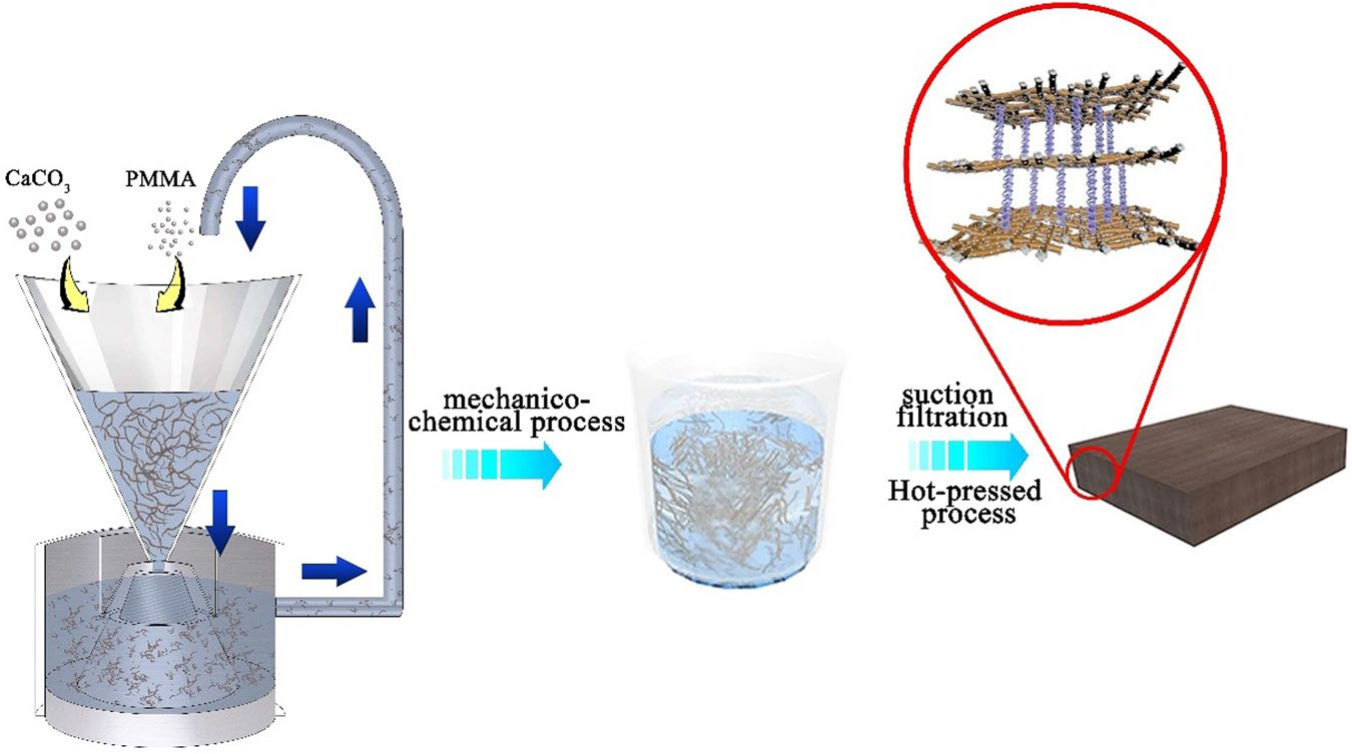
Schematic illustration of fabrication of NLC/CaCO_3_/PMMA composite.

The model of resultant NLC/CaCO_3_/PMMA composite was proposed as shown in Fig. 4. Firstly, the lignocellulose was broken, refined to nanoscale and its specific surface area was increased by mechanical force in the colloid grinder. Correspondingly, the crystallinity of cellulose in the lignocellulose declined, the lattice defects in the crystal structure caused the lattice displacement, and the system temperature increased at the same time. At this stage, the free energy of lignocellulose surface increased. Then, the intrant Ca and PMMA were adhered to the NLC surface by hydrogen bonding and electrostatic adsorption force. Finally, after redundant water of NLC/CaCO_3_/PMMA suspension was filtered, the composites were hot-pressed at 220 °C, 2.5 MPa and cured into the layered board. NLC platelets with CaCO_3_ nanoparticles were adhered by molten PMMA through strong hydrogen bonds.

**Figure 5:**
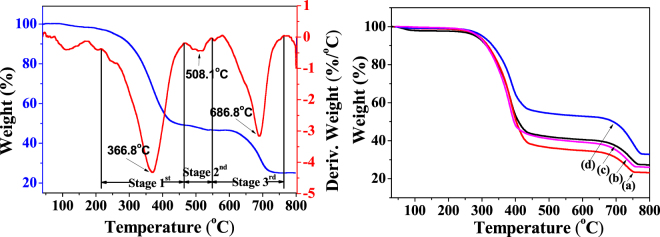
TGA and DTG curves of the NLC/CaCO_3_/PMMA composite and TGA of the NLC/CaCO_3_/PMMA composite with different concentrations of CaCO_3_: (**a**) 2wt.%, (**b**) 5 wt.%, (**c**) 8 wt.%, and (**d**) 11 wt.%, respectively.

The thermal stability of the NLC and NLC/CaCO_3_/PMMA composites was investigated by TGA and DTA, as shown in Fig. 5. The TGA curve of the NLC exhibited a small weight loss in the region from room-temperature to 100 °C due to the loss of adsorbed water (Fig. 5a). This weight loss was accompanied by the weak endothermic peak at 110 °C in the DTA curve. It was obvious that the weight loss mainly takes place at three stages: the first one (from 220 to 460 °C) was probably caused by thermal degradation of NLC, and the other (from 460 to 540 °C) was probably caused by the decomposition of NLC in the composites^40,41^. Weight loss at 580–740 °C was probably attributed to the degradation of CaCO_3_^42^. The TGA curves of the NLC/CaCO_3_/PMMA composites displayed a similar weight loss (Fig. 5a–d), compared with NLC. The total weight losses of the NLC/CaCO_3_/PMMA composites were ~76.8%, 74.0%, 72.8%, and 67.2% from room temperature to 600 °C corresponding to the content of CaCO_3_ of 2 wt.%, 5 wt.%, 8 wt.%, and 11wt.%, respectively (Fig. 5a–d). The NLC were mainly composed of lignin, cellulose, and hemicellulose, which were completely degraded to water, carbon dioxide (CO_2_) and ash under calcination at high temperature. In addition, the quantity of the lignocelluloses in each sample was certain. Therefore, the relatively high concentration of CaCO_3_ displayed the high thermal stability of the composites.

**Figure 6:**
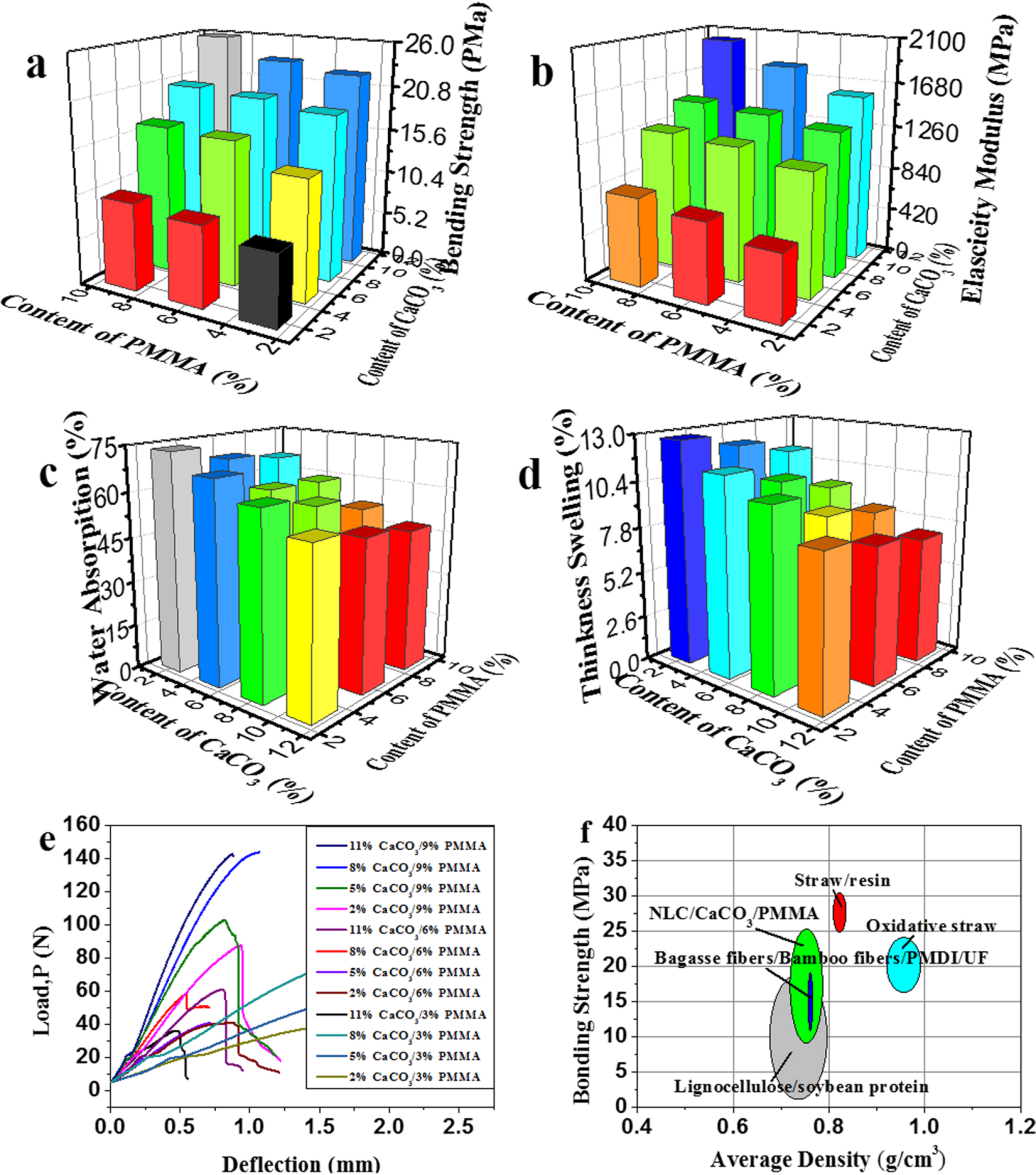
Bending strength (**a**), elasticity modulus (**b**), water absorption (**c**) thickness swelling (**d**) and bending stress-strain curves (**e**) of the hot-pressed NLC/CaCO_3_/PMMA composites with the concentrations of CaCO_3_ and PMMA from 2–11 wt.% and 3–9 wt.%, (**f**) Mechanical properties of NLC/CaCO_3_/PMMA composites compared with other related composites.

Bending strength and elasticity modulus of the produced composites varied from 8.1 to 25.8 MPa and 624.7 to 2011.28 MPa (Fig. 6a,b). It can be seen from Fig. 6a,b that the bending strength and elasticity modulus of the boards differ significantly with the content of CaCO_3_ and PMMA; the mechanical strength of the boards enhanced as the content of CaCO_3_ and PMMA increased. The reason for this behavior is attributed to the interconnectivity of NLC/CaCO_3_ composites through PMMA. If the PMMA adhesive contained very lower level, the NLC/CaCO_3_ composite layers tend to aggregate. The interaction between anionic NLC/CaCO_3_ composites was relatively weak. In addition, NLC/CaCO_3_ composites were quite rigid, high content of calcium carbonate can endow a higher rigidity of the composites.

Figure 6c,d showed the values of the water absorption and thickness swelling for the composites, which vary depending upon the filler loading, as shown in Fig. 6c,d, the thickness swelling and water absorption of the composites increased with increasing filler loading, but were nevertheless very low as compared with the control samples, because the matrix PMMA were hydrophobic, whereas the control samples were hydrophilic.

The three-point bending stress-strain curves of the NLC/CaCO_3_/PMMA composites with the content of CaCO_3_ and PMMA were plotted in Fig. 6e. In this study, the NLC/CaCO_3_/PMMA composites were indeed tougher with the increase of the content of CaCO_3_ and PMMA. The effect of combining polymer with lignocellulose-based materials can be seen in Fig. 6f, which is a material properties map for non-resin and resin lignocellulose-based materials^28,43–45^. On the horizontal axis, the modulus is a measure of the stiffness, while the vertical axis represents the density. At the upper left of the diagram is the realm of the ‘lightweight and high strength’, the macroscopic mechanical properties of the NLC/CaCO_3_/PMMA composite are superior to some non-resin and resin lignocellulose-based materials in the approximation density. To consider the pH increase and its impact on the NLC/CaCO_3_/PMMA composite, the strength of NLC/CaCO_3_/PMMA composite synthesized at different PH was tested (Figure S1). When water PH is higher than 7.1, the water of cation adsorption with negatively charged groups on the surface of the NLC, the fiberboard surface formed by cationic layer and prevent the electrostatic adsorption of NLC and calcium carbonate leads to a decline in the intensity of fiberboard.

**Figure 7:**
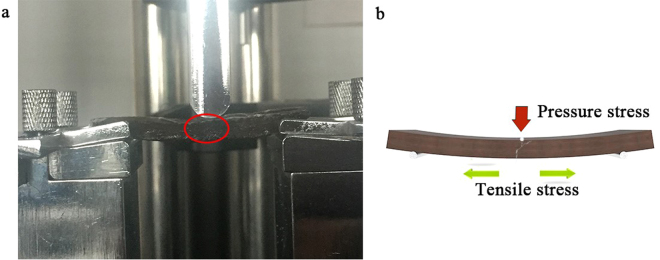
Lower surface fracture form (**a**) and lower surface ideal fracture form (**b**) of NLC/CaCO_3_/PMMA composites.

Based on experimental observation and the phenomenology, the bending failure modes of different types of the composites were analyzed. It was summarized that the mainly pattern of bending destroys was the snap of lower surface. For the three-point bending experiment, the maximum positive stress appears on the upper and lower surface of the beam loading point, the upper surface was subjected to pressure stress, and the lower surface was subjected to tensile stress. The tensile properties of the composites were less than the compressive resistance, therefore, when the maximum tensile stress of the lower surface of the beam exceeded the tensile strength of the composites, the lower surface of the loading point of the experimental beam started to break, and then it extended along the cross section that was approximately perpendicular to the axis. Finally, the experimental beam basically lost its bearing capacity, as showed in Fig. 7a. Due to the prepared composites were heterogeneous, the breaking point of the experimental beam was not always at the loading point, usually near the loading point. A small section of fault line that was approximately perpendicular to the axis was first formed at the bottom edge of the experimental beam, then the crack gradually turned, and it extended towards the loading point, which was about 45° in the direction of the axis, as showed in the Fig. 7b.

## Discussion

In summary, we reported the synthesis of NLC/CaCO_3_/PMMA composites using different concentrations of CaCO_3_ and PMMA by the mechano-chemical and hot-pressed method. The formation mechanism of the composites from NLC, PMMA and CaCO_3_ was proposed and discussed. Furthermore, when the content of CaCO_3_ and PMMA reached a certain level, activity of CaCO_3_ and PMMA was mainly as aggregates in the composites.

The mechanical, dimensional stability, and thermal properties of the composites enhanced as the filler loading increased. As the filler loading increased, the strong interfacial bonding between the filler and the NLC matrix caused the Bending strength and elasticity modulus of the composites to be increased, and this strong interfacial bonding resulted in a decrease in the number of micro voids, causing improved dimensional stabilities and water absorption behaviors. These materials proved to be good candidates to be used as underlay materials on floor applications. The developed composite materials present some promising properties, although reinforcement strategies to reach higher stiffness and strength could be needed for specific applications.

## Methods

### Materials

Lignocellulose based on softwood was obtained in a dried form, poly (methyl methacrylate) was supplied by Sinopharm Chemical Reagent Co., Ltd, calcium carbonate nanoparticle (nano-CaCO_3_) were purchased from Shanghai Boylechem Co., Ltd. The lignocellulose presents average diameter was about 20 μm, and the length was hundreds of microns to millimeters. The calcium carbonate nanoparticle presents a particle size of 5–50 nm.

### Fabrication of NLC/PMMA/CaCO_3_ composites

The lignocellulose suspension mixed with adjusting the content of CaCO_3_ (2–11 wt.%) and PMMA (3–9 wt.%) were simultaneously added rapidly to a colloid grinder with rotor speed set at around 2880 rpm and mechanico-chemical manufactured for 6 h. Lignocellulose/PMMA/CaCO_3_ suspension was fed into the colloid grinder continuously through a loop consisting of a peristaltic pump and plastic tubing. Finally, after redundant water of NLC/PMMA/CaCO_3_ suspension was filtered, the composites were hot-pressed at 220 °C, 2.5 MPa for 30 min and cured into the layered board. One can calculate the content of CaCO_3_ and PMMA to prepare the composite, and the data were listed in Table 1. For instance, Sample 7 was the lignocellulose board obtained with the content of CaCO_3_ (8 wt.%) and PMMA (6 wt.%).

**Table 1:** The content of CaCO_3_ and PMMA in the NLC/PMMA/CaCO_3_ composites.

Sample	density (g/cm^3^)	content of CaCO_3_ (wt.%)	content of PMMA (wt.%)
1	0.73	2	3
2	0.74	5	3
3	0.68	8	3
4	0.76	11	3
5	0.71	2	6
6	0.70	5	6
7	0.73	8	6
8	0.67	11	6
9	0.72	2	9
10	0.74	5	9
11	0.76	8	9
12	0.75	11	9

### Characterizations

The surface morphology of the composites was studied using Scanning Electron Microscopy (SEM, FEI, Quanta 200, USA). The high-resolution transmission electron microscopy (HRTEM) images from the Tecnai G2 F20 were used to obtain crystallographic information. X-ray diffraction (XRD) patterns were measured in 2θ range from 10° to 80° at a scanning speed of 5°/min on a Bruker D8 Advance with Cu-Kα radiation (λ = 1.5409 Å) diffraction meter. Fourier-transform infrared (FT-IR) spectroscopic measurements were carried out on a Nicolet 5700 spectrophotometer. X-ray photoelectron spectroscopy (XPS) was carried out on a ThermoFisher K-Alphato characterize the valence state of elements and depth compositional profiles of films. Thermal stabilities were determined using a TG analyzer (STA 449 F3, NETZSCH) with a heating rate of 10 °C min^−1^ in a N_2_ environment from 30 to 800 °C. The three-point bending tests were evaluated using a RegerRGM-6010T.

### Fracture testing

The composite fiberboard was measured 10 mm × 60 mm × 2.5 mm, the fracture tests were performed using a three-point bending fixture mounted on a miniature loading stage (Reger RGM-6010T) with a span length of 30 mm, and the specimens were loaded at a rate of 0.05 mm s^−1^ up to failure. For each composition, more than 10 samples were tested, from which the mean and standard deviation was calculated.

### Dimensional stability tests

The thickness swelling and water absorption tests were conducted according to ASTM D 1037–99. To determine the water absorption, specimens (measuring 6 mm in thickness, 60 mm wide and 90 mm long) from the different materials were immersed in distilled water, at 23 ± 1 °C and atmospheric pressure, for different time periods (up to 11 days). At each testing time, samples were removed from the water, patted dry and then weighed. Each value obtained represented the average of five samples. The water absorption was calculated according to:

1 $${\rm{Water}}\,{\rm{absorption}}\,( \% )=\frac{{W}_{a}-{W}_{b}}{{W}_{b}}\times 100$$

where W_a_ = weight of the specimen after being immersed for a certain period of time and W_b_ = weight of the same specimen before immersion (g).

To determine thickness swelling after immersion, the thickness of each immersed specimen was measured in two different points using a digital micrometre (±0.01 mm). The thickness swelling was calculated as follows:

2 $${\rm{Thickness}}\,{\rm{swelling}}\,( \% )=\frac{{T}_{2}-{T}_{1}}{{T}_{1}}\times 100$$

where T_2_ = thickness of the specimen after immersion and T_1_ = thickness of the same specimen before immersion. Three specimens per each condition were measured.

## Electronic supplementary material

Supplementary Information
