# Author Correction: The properties of fibreboard based on nanolignocelluloses/CaCO_3_/PMMA composite synthesized through mechano-chemical method

**DOI:** 10.1038/s41598-018-30921-9

**Published:** 2018-08-17

**Authors:** Yipeng Chen, Tailong Cai, Baokang Dang, Hanwei Wang, Ye Xiong, Qiufang Yao, Chao Wang, Qingfeng Sun, Chunde Jin

**Affiliations:** 1 School of Engineering, Zhejiang A & F University, Hangzhou, Zhejiang Province 311300 PR China; 2 Key Laboratory of Wood Science and Technology, Hangzhou, Zhejiang Province 311300 PR China

Correction to: *Scientific Reports* 10.1038/s41598-018-23497-x, published online 23 March 2018

The Acknowledgements section in this Article is incomplete.

“This research was supported by Special Fund for Forest Scientific Research in the Public Welfare (Grant No. 201504501), Zhejiang Xinmiao Talents Program (Grant No. 2018R46), Key Laboratory of Bio-based Material Science & Technology (Northeast Forestry University), Ministry of Education (Grant No. SWZCL2016-3) and Zhejiang Xinmiao Talents Program (Grant No. 2017R412011).”

should read:

“This research was supported by Special Fund for Forest Scientific Research in the Public Welfare (Grant No. 201504501), Zhejiang Xinmiao Talents Program (Grant No. 2018R412047), Key Laboratory of Bio-based Material Science & Technology (Northeast Forestry University), Ministry of Education (Grant No. SWZCL2016-3) and Zhejiang Xinmiao Talents Program (Grant No. 2017R412011).”

