# Supplementary material for: The properties of fibreboard based on nanolignocelluloses/CaCO3/PMMA composite synthesized through mechano-chemical method
Source: Sci Rep. 2018 Mar 23;8:5121. doi: 10.1038/s41598-018-23497-x (PMC5865124; doi:10.1038/s41598-018-23497-x)
Supplement: Supplementary file 1 — Supplementary Information [file 41598_2018_23497_MOESM1_ESM.docx]

Correspondence and requests for materials should be addressed to: Q.F. Sun ([qfsun@zafu.edu.cn](mailto:qfsun@zafu.edu.cn)); C.D. Jin (zafujincd@163.com)

**Title:** **The properties of fibreboard based on nanolignocelluloses/CaCO_3_/PMMA composite synthesized through mechano-chemical method**

Author names: Yipeng Chen^1^ ^&^, Tailong Cai^1^ ^&^, Baokang Dang^1^, Hanwei Wang^1^, Ye Xiong^1^, Qiufang Yao^1^, Chao Wang^1^, Qingfeng Sun^1,2*^, Chunde Jin^1,2*^

^&^ These authors contributed equally to this work.

Affiliations: 1. School of Engineering, Zhejiang A & F University, Hangzhou, Zhejiang Province, 311300, PR China; 2. Key Laboratory of Wood Science and Technology, Zhejiang Province, 311300, PR China

Figure S1. The strength of NLC/CaCO_3_/PMMA composite synthesized at different PH was tested.
